# Implication of Low HDL-c Levels in Patients with Average LDL-c Levels: A Focus on Oxidized LDL, Large HDL Subpopulation, and Adiponectin

**DOI:** 10.1155/2013/612038

**Published:** 2013-10-24

**Authors:** Filipa Mascarenhas-Melo, José Sereno, Edite Teixeira-Lemos, Daniela Marado, Filipe Palavra, Rui Pinto, Petronila Rocha-Pereira, Frederico Teixeira, Flávio Reis

**Affiliations:** ^1^Laboratory of Pharmacology & Experimental Therapeutics, IBILI, Faculty of Medicine, Sub-Unit 1 (Pólo III), University of Coimbra, 3000-548 Coimbra, Portugal; ^2^ESAV and Educational Technologies and Health Study Center, Polytechnic Institute of Viseu, 3504-510 Viseu, Portugal; ^3^Internal Medicine Department, General Hospital, University and Hospital Centre of Coimbra, 3041-801 São Martinho do Bispo, Portugal; ^4^Neurology Department, General Hospital, University and Hospital Centre of Coimbra, 3041-801 São Martinho do Bispo, Portugal; ^5^Pharmacology and Pharmacotoxicology Unit, Faculty of Pharmacy, University of Lisbon, 1649-003 Lisbon, Portugal; ^6^Research Centre for Health Sciences, Beira Interior University, 6201-001 Covilhã, Portugal

## Abstract

To evaluate the impact of low levels of high density lipoprotein cholesterol (HDL-c) on patients with LDL-c average levels, focusing on oxidative, lipidic, and inflammatory profiles. Patients with cardiovascular risk factors (*n* = 169) and control subjects (*n* = 73) were divided into 2 subgroups, one of normal HDL-c and the other of low HDL-c levels. The following data was analyzed: BP, BMI, waist circumference and serum glucose Total-c, TGs, LDL-c, oxidized LDL, total HDL-c and subpopulations (small, intermediate, and large), paraoxonase-1 (PON1) activity, hsCRP, uric acid, TNF-**α**, adiponectin, VEGF, and iCAM1. In the control subgroup with low HDL-c levels, significantly higher values of BP and TGs and lower values of PON1 activity and adiponectin were found, versus control normal HDL-c subgroup. However, differences in patients' subgroups were clearly more pronounced. Indeed, low HDL-c subgroup presented increased HbA1c, TGs, non-HDL-c, Ox-LDL, hsCRP, VEGF, and small HDL-c and reduced adiponectin and large HDL. In addition, Ox-LDL, large-HDL-c, and adiponectin presented interesting correlations with classical and nonclassical markers, mainly in the normal HDL-c patients' subgroup. In conclusion, despite LDL-c average levels, low HDL-c concentrations seem to be associated with a poor cardiometabolic profile in a population with cardiovascular risk factors, which is better evidenced by traditional and nontraditional CV biomarkers, including Ox-LDL, large HDL-c, and adiponectin.

## 1. Introduction

Dyslipidemia is recognized as one of the major risk factors for the development of cardiovascular disease (CVD), which is a major clinical problem worldwide. Large prospective cohort studies, such as the Framingham Heart Study and the Seven Countries Study, have been recognizing the importance of reducing major risk factors, including cholesterol levels, in particular low-density lipoprotein cholesterol (LDL-c), as a pivotal strategy to prevent the development/evolution of cardiovascular disease and related events [1–3]. However, it is now accepted that the current lipid-lowering therapies, in particular those directed to reduce LDL-c levels, such as statins, are insufficient to prevent part of the cardiovascular events; indeed, residual cardiovascular risk remains elevated even in clinical trials in which LDL-c levels have been aggressively reduced [4–6]. In fact, it has been accepted that a considerable proportion of cardiovascular events occur in individuals who do exhibit normal LDL-c levels, and there is a residual cardiovascular risk that has been the focus of a great deal of interest [7–10]. Furthermore, this fact reinforces the idea that traditional risk factors, including lipidic, might not tell the whole story about CVD progression and prevention of CV events and, thus, there has been an increasing interest in identifying novel biomarkers that might improve the global risk prediction of CVD [11, 12]. In addition to the critical role that LDL-c plays, several lines of evidence have shown the contribution of other lipid fractions/components, such as oxidized LDL (Ox-LDL) and high-density lipoprotein cholesterol (HDL-c), to overall cardiovascular health [3, 13–15].

LDL oxidation is associated with coronary artery disease (CAD) as well as with other disorders, as recently emerged from experimental and clinical studies [16–20]. Concerning the CAD, Ox-LDL is a promoter of key steps in the onset and evolution of atherosclerosis, including stimulation of monocyte infiltration and smooth muscle cell migration and proliferation; conversely, high levels of HDL-c prevent the development of atherosclerosis and CAD, in particular due to the transport of reserve cholesterol and the inhibition of Ox-LDL induced monocyte infiltration; Ox-LDL and HDL are indeed antagonists in the development of CVD [21]. Removal and/or inactivation of circulating Ox-LDL has been increasingly viewed as a promising therapeutic strategy against atherosclerosis, but more research is mandatory to clarify some discrepant data [22, 23]. Concerning the management of HDL-c, clinical and epidemiologic data illustrate the need to expand the scope of therapies to reduce the residual cardiovascular risk associated with low HDL-c levels, even when LDL-c is managed successfully [24–26]. In fact, low plasma levels of HDL-c have been largely recognized as a risk factor for coronary heart disease (CHD) [27, 28].

It has been suggested that monitoring the type of HDL particles, which carry distinct and specific proteins or lipids and are differentiated by density and size (large, intermediate and small), rather than their total quantity, is a more reasonable way of determining the CV risk, suggesting that different subpopulations may have a different role in reverse cholesterol transport (RCT) and CVD risk protection [29]. Actually, some recent studies have been reporting that large HDL levels are reduced in patients with CAD compared to healthy subjects and inversely related to both disease severity and progression of coronary lesions [30]. Although the most widely known mechanism behind the antiatherogenic function of HDL is the RCT, other important protective properties have been described, including anti-inflammatory, antioxidant, antithrombotic and vasorelaxant [31–34]. Although the benefit of high HDL-c contents appears to be obvious, most clinical trials that aimed at increasing HDL-c concentrations failed to generate convincing results. Therefore, the question arises as to whether the quantification of HDL-c level or perhaps rather more the HDL function and subpopulations is of considerable therapeutic relevance [35]. In fact, variations in HDL subfraction levels and functions have been observed in CVD populations, suggesting that large HDL particles are inversely associated with atherosclerosis development while small HDL particles are positively connected with CVD, which is also observed for Ox-LDL contents [36–39]. These considerations indicate that beside the measurement of standard lipids (such as HDL-c and LDL-c levels), the measurement of specific HDL subfractions and Ox-LDL might help to better evaluate the risk of cardiovascular events in specific populations.

Improved characterization of the impact of low-HDL-c levels in populations with normalized LDL-c concentrations and the relevance of HDL subpopulations and Ox-LDL contents will be an important step to develop strategies better directed to reduce dyslipidemia-associated cardiovascular risk. Thus, this study aimed to evaluate the influence of low HDL-c levels on the cardiometabolic profile of patients with cardiovascular risk factors but average LDL-c contents, using both traditional and new nontraditional markers, including Ox-LDL, HDL subpopulations, and inflammatory and angiogenesis mediators.

## 2. Materials and Methods

### 2.1. Subjects and Ethical Consideration

Two hundred and forty-two subjects were enrolled in the study, aged 33 to 75 years, divided in two major groups: control volunteers and patients with cardiovascular risk factors (designed as control and as patients, resp.). The control volunteers were randomly recruited during the performance of routine laboratory analysis in a clinical laboratory and were selected after not expressing any diagnosis or taking medication for cardiovascular disease and no family history of CVD. The group included 73 subjects, 39 males and 34 females. The patients group involved 169 volunteers, 88 males and 81 females, defined as having cardiovascular risk factors in terms of previous diagnosis and/or pharmacological treatment for hypertension and/or for type 2 diabetes mellitus (T2DM) and/or for dyslipidemia. T2DM was diagnosed in the Diabetes and Metabolic Diseases Unit from the Coimbra Hospital Centre (EPE), according to the European Guidelines. Patients were recruited during the performance of routine laboratory analysis on the basis of previous diagnosis and/or treatment for hypertension and dyslipidemia, performed according to the International Society of Hypertension/World Health Organization and the Seventh Joint National Committee on Hypertension and National Cholesterol Education Program-Adult Treatment Panel III (NCEP-ATP III) for hypertension and dyslipidemia, respectively. Other cardiovascular disorders of the patients' population include coronary artery and heart disease, ischemic heart failure, angina pectoris, arrhythmias, atrial fibrillation, cardiac valvulopathies, and peripheral vascular disease. Five patients reported a previous cerebrovascular event and other 5 a stroke episode. The patients were under the following medication: (a) insulin and/or oral antidiabetic drugs (OAD)—60.95%: in detail, 41 patients with insulin, 37 patients with sulfonylureas, 47 patients with biguanides, 51 patients with modulators of incretins, and 10 patients with alpha-glucosidase inhibitors; (b) lipid-lowering drugs—63.91%, being 90 patients under statins therapy, 21 under fibrates, 3 using an inhibitor of cholesterol absorption (ezetimibe), and 1 patient with omega-3; (c) antihypertensive drugs—70.41%, in particular 73 patients with diuretics (44 thiazides and analogues, 26 of the loop, 3 potassium-sparing), 47 with angiotensin-converting-enzyme inhibitors, 56 with angiotensin II receptor antagonists, 35 with calcium channel blockers, 32 with beta blockers, and 5 with central alpha-2 agonists. Some of the patients were under combined therapies.

The control subject did not take any drug for cardiovascular disease. Pregnant women and people with age <16 or >75 years were excluded. Each group was divided into two subgroups of normal HDL-c and of low-HDL-c levels, using the cutoffs of 1.03 mmol/L for men and 1.29 mmol/L for women, according to NCEP-ATP III guidelines. The smoking habits of the populations were (i) nonsmokers—46 normal-HDL-c control volunteers (90.20%), 19 low-HDL-c control volunteers (86.36%), 107 normal-HDL-c patients (89.92%), and 46 low-HDL-c patients (92.00%); (ii) ≤10 cigarettes a day—5 control-HDL-c control volunteers (9.80%), 3 low-HDL-c control volunteers (13.64%), 8 normal-HDL-c patients (6.72%), and 3 low-HDL-c patients (6.00%); (iii) >10 cigarettes a day—0 normal-HDL-c control volunteers (0.00%), 0 low-HDL-c control volunteers (0.00%), 4 normal-HDL-c patients (3.36%), and 1 low-HDL-c patients (2.00%). The study was performed in agreement with the code of ethics of the World Medical Association (Declaration of Helsinki) for human studies and received authorization from the local ethics committee, as well as from all the participants by signing a written informed consent.

### 2.2. Data and Blood Collection

The following data was obtained from each subject by trained personnel: weight and height (without shoes and wearing light outdoor clothing) were measured in order to calculate body mass index (BMI); waist circumference (WC) was assessed, as well as systolic and diastolic blood pressure (SBP and DBP) the latter of which was assessed in the sitting position after a 5 min rest. Blood samples were collected by venipuncture from the subjects after an overnight fasting period, via both EDTA-containing tubes and tubes without anticoagulant, in order to obtain plasma, buffy-coat, and serum, and processed within 2 hours of collection. Aliquots were immediately stored at −80°C until assayed.

### 2.3. Assays

#### 2.3.1. Lipid Profile

Serum total cholesterol (Total-c), HDL cholesterol (HDL-c), LDL cholesterol (LDL-c), and triglycerides (TGs) were analysed on a Hitachi 717 analyser (Roche Diagnostics) using standard laboratorial methods. Total-c reagents and TGs kit were obtained from bioMérieux sa (Lyon, France). HDL-c Plus and LDL-c Plus tests were obtained from F. Hoffmann-La Roche Ltd. (Roche Diagnostics Div., Basel, Switzerland). Serum glucose levels were measured using a Glucose Oxidase commercial kit (Sigma, St. Louis, Mo, USA). Plasma concentration of Ox-LDL was evaluated by using a standard commercial enzyme-linked immunoassay (Oxidized LDL ELISA, Mercodia, Uppsala, Sweden). 

#### 2.3.2. HDL Subpopulations Assay

Subpopulations were separated and quantified using a Lipoprint kit from Quantimetrix Corp. (Redondo Beach, CA, USA). The assay involves a polyacrylamide gel electrophoresis assay and a complete Lipoprint System for data acquisition and quantification of large, intermediate, and small subpopulations of HDL. 

#### 2.3.3. PON1 Paraoxonase Activity

This was assessed spectrophotometrically and expressed in nmol of pnitrophenol/mL/min. In brief, paraoxonase activity was measured by adding serum to 1 mL Tris/HCl buffer (100 mmol/L, pH 8.0) containing 2 mmol/L CaCl_2_ and 5.5 mmol/L paraoxon (O,O-diethyl-O-p-nitrophenylphosphate; Sigma Chemical Co.). The rate of generation of p-nitrophenol was determined at 412 nm, 37°C, via the use of a continuously recording spectrophotometer (Beckman DU-68). 

### 2.4. Serum Inflammatory and Angiogenic and Endothelial Markers

Serum adiponectin, TNF-*α*, and VEGF contents were assessed using Quantikine enzyme-linked immunoassays kits from R&D Systems (Minneapolis, USA); serum intercellular adhesion molecule 1 (iCAM1) levels were evaluated by using an Elisa kit from Abcam (Cambridge, MA, USA); high-sensitivity C-reactive protein (hsCRP) was evaluated by immunoturbidimetry, using commercially available kits (CRP (latex) High-Sensitivity, Roche Diagnostics); uric acid was analysed on a Hitachi 717 analyser (Roche Diagnostics) using standard laboratory methods.

### 2.5. Statistical Analysis

Statistical analysis was performed by using the IBM Statistical Package for Social Sciences (SPSS) for Windows, version 20.0, (SPSS, Inc., Chicago, IL, USA). The distribution of continuous variables was analyzed using Kolmogorov-Smirnov tests to assess significant departures from normality. Results for normal distribution samples are presented as mean SD and lower and upper bound 95% confidence interval for mean; *P* values were obtained using independent samples *t*-test. Results for non-normal distribution samples are presented as median SD and interquartile range; *P* values obtained using Mann-Whitney test. The association between categorical variables was analyzed using Pearson's test. Statistical significance was accepted at *P* less than 0.05.

## 3. Results

### 3.1. Anthropometric Data

The demographic and anthropometric data of controls and patients are summarized in Table 1. Both populations were divided according to the HDL-c levels: normal-HDL-c (men > 1.03 mmol/L and women > 1.29 mmol/L) and low HDL-c (men ≤ 1.03 mmol/L and women ≤ 1.29 mmol/L), which were then compared (normal HDL-c versus low HDL-c) for each population under study (control and patients). Seventy-three control volunteers were enrolled in the study: 51 (69.86%) normal HDL-c and 22 (30.14%) low HDL-c. One hundred and sixty-nine patients were recruited: 119 (70.41%) normal HDL-c and 50 (29.59%) low HDL-c. Normal and low HDL-c groups presented no differences concerning age and obesity (BMI and waist circumference), in both study populations (Table 1). Blood pressure (systolic and diastolic) was significantly higher in low HDL-c when compared with normal HDL-c in the control group, while no differences were found between the subgroups of patients. Concerning the glucidic profile, no differences were found for glycemia and HbA1c between normal and low HDL-c subgroups of control subjects, while a significantly increased value of HbA1c was found in the subgroups of patients with low HDL-c levels when compared with the normal HDL-c subgroup of patients (Table 1).

### 3.2. Classical Lipid Profile and Oxidized LDL Content

The subjects entering in the control group were without any cardiovascular therapy, including lipid-lowering agents, while the majority of subjects from the patients group were under antidyslipidemic therapy, which justify some of the data obtained for the classic lipid profile. In the control group, lower values of Total-c were found in the low HDL-c subgroup when compared with normal HDL-c one, accompanied by significantly increased contents of TGs. In addition, while no differences were found for LDL-c, Ox-LDL, and non-HDL-c, there were significantly higher values of Total-c/HDL-c and LDL-c/HDL-c ratios (Table 2). However, the differences between the subgroups of patients (normal versus low-HDL-c levels) were more expressive. Indeed, the subgroups of patients with low-HDL-c levels presented a trend to increased values of Total-c and LDL-c, but statistically significant higher of TGs, Ox-LDL, and non-HDL-c, as well as of Total-c/HDL-c and LDL-c/HDL-c ratios (Table 2). 

### 3.3. HDL Subpopulations and Paraoxonase Activity

**Figure 1:**
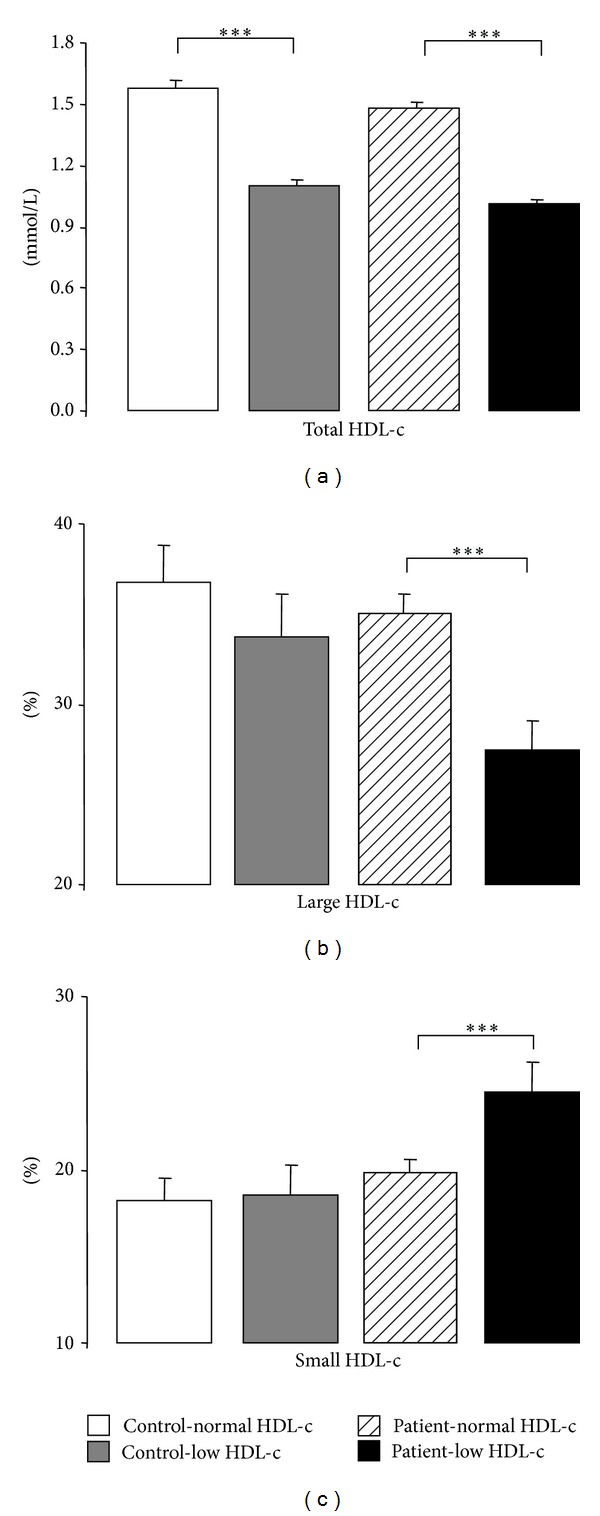
Serum total-HDL-c (a), large HDL-c (b), and small HDL-c (c), in the study groups. Results are presented as mean ± SEM. ***P < 0.001.

Regarding the content of HDL subpopulations, despite the lower levels in both low HDL-c groups (which is obvious by definition of the study groups) (Table 2 and Figure 1(a)), only in the patients population there was a significantly decreased percentage of large HDL-c and increased percentage of small HDL-c, while no differences of HDL subpopulations percentages were found between the two subgroups of controls (normal versus low HDL-c) (Table 2 and Figures 1(b) and 1(c), resp.). Concerning PON1 activity, in the control group, there was a reduced value in the low HDL-c subgroup, while unchanged values were encountered between the two subgroups of patients (Table 2).

### 3.4. Markers of Inflammation, Angiogenesis, and Endothelial Lesion

**Figure 2:**
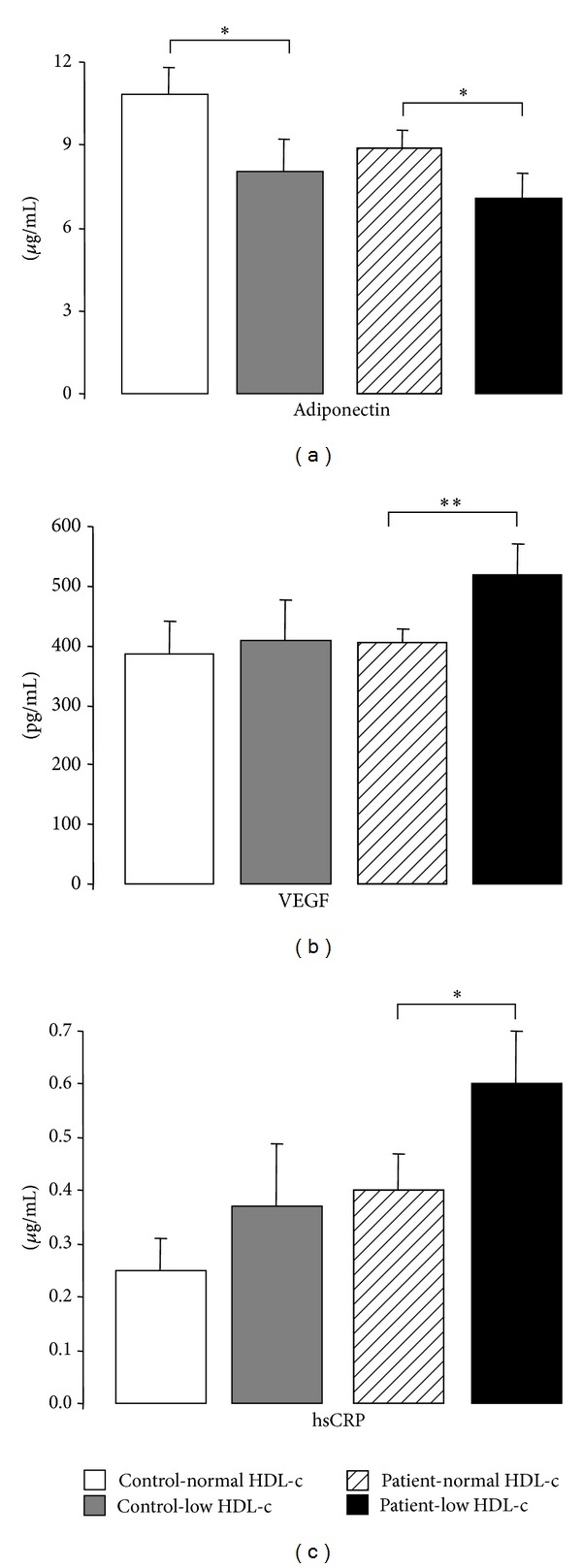
Serum adiponectin (a), VEGF (b), and hsCRP (c) levels, in the study groups. Results are presented as mean ± SEM. *P < 0.05 and **P < 0.01.

Regarding other putative markers of cardiovascular disease, in the control individuals, the reduced content of HDL-c was associated only with a significantly reduced concentration of adiponectin (Figure 2(a)), when compared with controls subjects with normal HDL-c levels; all the other parameters were unchanged, including hsCRP, TNF-*α*, uric acid, iCAM-1, and VEGF (Table 2 and Figure 2). However, in the patients' population, the reduced content of HDL-c was associated not only with an additional reduction of adiponectin (Figure 2(a)) but also with significantly increased concentrations of VEGF and hsCRP (Figures 2(b) and 2(c), resp.), when compared with patient subgroup with normal HDL-c levels (Table 2 and Figure 2). 

### 3.5. Analysis of Correlations between Markers of CV Risk in Patients Subgroups

**Figure 3:**
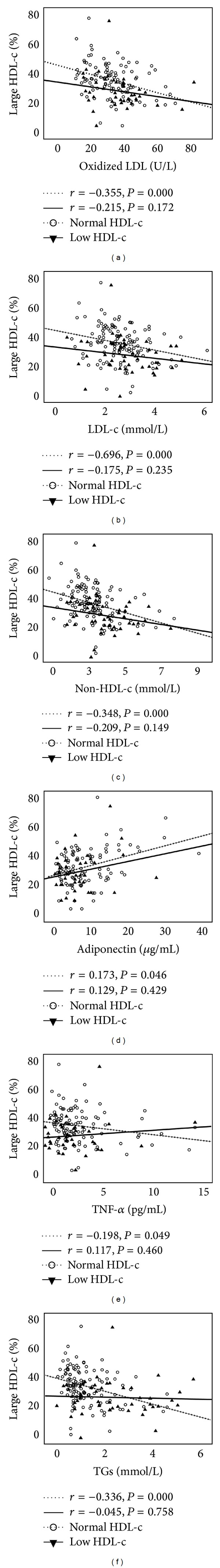
Main correlations in normal and low-HDL-c patients. Correlation between large HDL-c and Ox-LDL (a), LDL-c (b), non-HDL-c (c), adiponectin (d), TNF-*α* (e), and TGs (f).

**Figure 4:**
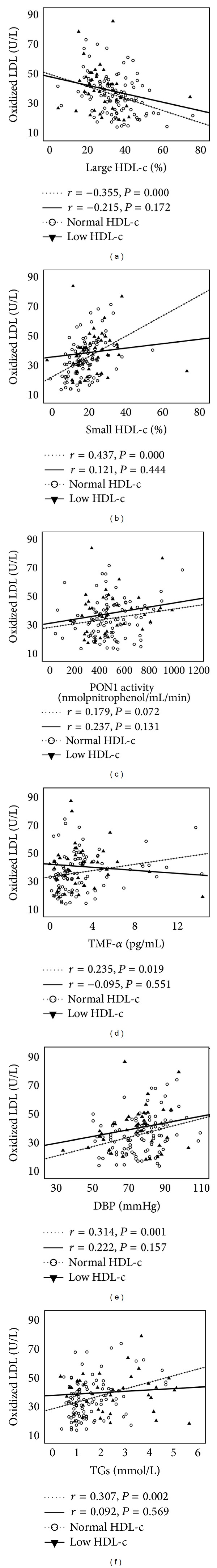
Main correlations in normal and low HDL-c patients. Correlation between Ox-LDL-c and large HDL-c (a), small HDL-c (b), PON1 activity (c), TNF-*α* (d), DBP (e), and TGs (f).

**Figure 5:**
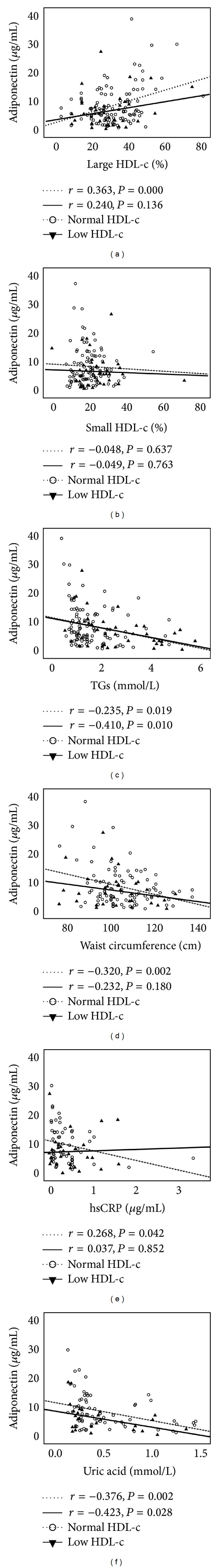
Main correlations in normal and low-HDL-c patients. Correlation between adiponectin and large HDL-c (a), small HDL-c (b), TGs (c), waist circumference (d), hsCRP (e), and uric acid (f).

The values of large-HDL in the normal HDL-c patients' subgroup were negatively and significantly correlated with Ox-LDL (*r* = −0.355, *P* = 0.000) (Figure 3(a)), LDL-c (*r* = −0.696, *P* = 0.000) (Figure 3(b)), non-HDL-c (*r* = −0.348, *P* = 0.000) (Figure 3(c)), TNF-*α* (*r* = −0.198, *P* = 0.049) (Figure 3(e)), and TGs (*r* = −0.336, *P* = 0.000) (Figure 3(f)) levels and positively and significantly correlated with adiponectin (*r* = 0.173, *P* = 0.046) (Figure 3(d)) but not in the low-HDL-c patients' subgroup (versus Ox-LDL: *r* = −0.215, *P* = 0.172; versus LDL-c: *r* = −0.175, *P* = 0.235; versus non-HDL-c: *r* = −0.209, *P* = 0.149; versus adiponectin: *r* = 0.129, *P* = 0.429; versus TNF-*α*: *r* = 0.117, *P* = 0.460; versus TGs: *r* = −0.045, *P* = 0.758) (Figure 3(a) to Figure 3(f), resp.). In addition, in the normal HDL-c patients' subgroup, Ox-LDL was negatively and significantly correlated with large HDL-c (*r* = −0.355, *P* = 0.000) (Figure 4(a)) and positively and significantly correlated with small HDL-c (*r* = 0.437, *P* = 0.000) (Figure 4(b)), TNF-*α* (*r* = 0.235, *P* = 0.019) (Figure 4(d)), DBP (*r* = 0.314, *P* = 0.001) (Figure 4(e)), and TGs (*r* = 0.307, *P* = 0.002) (Figure 4(f)), together with a trend to correlation with PON1 activity (*r* = 0.179, *P* = 0.072) (Figure 4(c)). These correlations showed statistically unchanged values in the low HDL-c patients' subgroup (versus large HDL-c: *r* = −0.215, *P* = 0.172; versus small HDL-c: *r* = 0.121, *P* = 0.444; versus PON1 activity: *r* = 0.237, *P* = 0.131; versus TNF-*α*: *r* = −0.095, *P* = 0.551; versus DBP: *r* = 0.222, *P* = 0.157; versus TGs: *r* = 0.092, *P* = 0.569) (Figure 4(a) to Figure 4(f), resp.). Finally, also in normal-HDL-c patients' subgroup, adiponectin was positively and significantly correlated with large HDL-c (*r* = 0.363, *P* = 0.000) (Figure 5(a)) and negatively and significantly correlated with TGs (*r* = −0.235, *P* = 0.019) (Figure 5(c)), waist circumference (*r* = −0.320, *P* = 0.002) (Figure 5(d)), hsCRP (*r* = −0.268, *P* = 0.042) (Figure 5(e)), and uric acid (*r* = −0.376, *P* = 0.002) (Figure 5(f)) but not in low HDL-c patients' subgroup (with the exception of TGs and uric acid) (versus large HDL-c: *r* = 0.240, *P* = 0.136; versus TGs: *r* = −0.410, *P* = 0.010; versus waist circumference: *r* = −0.232, *P* = 0.180; versus hsCRP: *r* = 0.037, *P* = 0.852; versus uric acid: *r* = −0.423, *P* = 0.028, resp.) (Figure 5). In opposition with large HDL-c, no significant correlation was found between adiponectin and small HDL-c in both normal (*r* = −0.048, *P* = 0.637) and low HDL-c (*r* = −0.049, *P* = 0.763) patients' subgroups (Figure 5(b)).

## 4. Discussion

The main finding of this study is that low HDL-c levels are associated with a poor cardiometabolic profile in a population of patients with cardiovascular risk factors, which is better diagnosed when analyzed in terms of nontraditional markers, including large HDL subpopulation, Ox-LDL, adiponectin, and VEGF. Although in a lesser extent, the impact of low-HDL-c levels is also manifest in the control population, viewed by an increase of blood pressure (SBP and DBP) and TGs concentration, and by a decrease of PON1 activity, adiponectin, and large HDL-c levels. However, when analyzing the patient population, the low HDL-c subgroup presents a notorious worse cardiometabolic profile when compared with normal HDL-c subgroup of patients, being the differences clearly more pronounced than those encountered in the control subjects. The impact of low-HDL-c levels is seen by some classical parameters but mainly by nonclassical markers. Indeed, patients with low concentration of HDL-c present increased contents of HbA1c, TGs, non-HDL-c, Ox-LDL, hsCRP, VEGF, and small HDL, as well as decreased concentration of adiponectin and of large-HDL.

Despite the recognition of an association between low levels of HDL-c with increased risk for CAD [40, 41], it has been suggested that a better indicator of HDL functionality may be their quality [42, 43], which depends on its subpopulation's type (large versus small) and constituents, including PON 1 activity [44, 45]. Our results are in agreement with this theory; indeed, the beneficial HDL profile found in normal HDL-c subgroups, relative to the corresponding low HDL-c ones, was reinforced by significantly increased content of large HDL-c and decreased of small HDL-c (specially in the patients population). Thus, low HDL-c values are associated with a seemingly less protective subpopulation typology. Genest et al. [46] reported that although 34% of patients with premature heart disease had LDL-c levels >160 mg/dL, more than half of the patients with premature heart disease (57%) had low HDL-c levels. Additionally, it has been reported that in patients with premature CAD the greatest risk factor is actually low HDL-c levels, though these individuals often possess high TG concentration as well [47]. These studies are in agreement with our results showing a poor cardiometabolic profile in the subgroups with low HDL-c levels, both being accompanied by increased amounts of triglycerides. 

Concerning the blood pressure, the values of both SBP and DBP in the control population are indeed higher when compared with the patients' population, no matter the normal or low HDL-c levels, which might be analyzed in terms of the antihypertensive therapy of the patients' population. In these subjects, medication is able to normalize blood pressure in both subgroups (normal and low HDL-c). However, in the control nonmedicated population, the subgroup with low DHL-c levels presents significantly increased values of both SBP and DBP. Several aspects related with HDL functionality might contribute to explain the differences of BP in the low versus normal HDL-c subgroups. In fact, as mentioned above, HDL has distinct properties that contribute to a healthy vasculature, such as antioxidant and anti-inflammatory action and inhibition of expression of cell adhesion molecules in endothelial cells, as well as antithrombotic and vasorelaxant effects, including the promotion of nitric oxide and prostacyclin release by vascular cells [31–34], which is expected to have a beneficial impact on arterial stiffness and blood pressure, in agreement with the suggestion of Woodman et al. [48]. In the presence of antihypertensive medication (as occurs in the patients' population) these differences were absent, but the data from the control nonmedicated subjects seems to be important per se. In fact, according to the South-West Seoul (SWS) study, performed in an elderly Korean population, prehypertension is not associated with increased risk of mortality, but individuals with high-normal blood pressure, when combined with low HDL-c, showed a significantly increased risk of all-cause mortality [49], reinforcing the relevance of our findings in this population of subjects with low-HDL-c levels and high-normal blood pressure, which were yet not diagnosed nor medicated for any cardiovascular disease, including for blood pressure and/or lipids.

In relation to the markers of inflammation, our study is in agreement with the study of Khan et al. [50] which has reported that a decrease in serum HDL levels and an increase in hsCRP values strongly predispose the risky individuals to an acute myocardial infarct (AMI) event; in addition, the reduction of serum total cholesterol does not prevent the risk of AMI. In addition, inflammation seems to have a deleterious impact on the antiatherogenic properties of HDL, suggesting that HDL function assessment is of particular importance when predicting CV risk in patients with chronic inflammatory diseases [51]. In our study, adiponectin levels also indicated an interesting association with the values of HDL cholesterol. Adiponectin is a novel adipocyte-specific protein which plays a role in the development of insulin resistance and atherosclerosis [52]. In our study, in both low-HDL-c subgroups the levels of adiponectin were decreased, which is in agreement with Fernandez et al. [53] which reported that individuals with low HDL-c concentrations present an increased risk for diabetes, as they show increased insulin resistance and lower levels of adiponectin. 

Inflammation and oxidative stress are key pathways in the development of atherosclerosis, with oxidized LDL being one of the major players in this process, together with several mediators of inflammation [13, 14, 54]. Oxidized LDL induces atherosclerosis by stimulating monocyte infiltration and smooth muscle cell migration and proliferation. It contributes to atherothrombosis by inducing endothelial cell apoptosis and thus plaque erosion, by impairing the anticoagulant balance in endothelium, stimulating tissue factor production by smooth muscle cells, and inducing apoptosis in macrophages [55]. HDL cholesterol levels are inversely related to risk of CAD and prevent atherosclerosis by reversing the stimulatory effect of oxidized LDL on monocyte infiltration [21, 56]. The HDL-associated enzyme paraoxonase inhibits the oxidation of LDL and its effects [44, 45, 57]. In our study, the levels of Ox-LDL are increased in the subgroup of patients with low HDL-c, although there are no changes in the values of PON1 activity. On the contrary, in the control population, a decreased PON1 activity was found in the subgroup with low HDL-c group, without changes on Ox-LDL contents. There seems to be a correlation between these three parameters, which most probably is related with the above-mentioned functionality (quality) of HDL in the control and patients populations, as well as with the previously reported effects of antidyslipidemic therapy, namely, statins, on Ox-LDL and PON1 activity [58, 59].

Endothelial dysfunction is thought to play a critical role in the development and progression of atherosclerosis and several recent studies have suggested that HDL exerts direct endothelial-protective effects, such as stimulation of endothelial production of the anti-atherogenic molecule nitric oxide, as well as antioxidant, anti-inflammatory, and antithrombotic effects [31–34]. Furthermore, it has been observed that HDL may stimulate endothelial repair processes, involving mobilization and promotion of repair capacity of endothelial progenitor cells [60]. In addition, VEGF has been viewed as a stimulating factor for the progenitor cells and the cell migration response [61]; in our study, although there were no significant changes in serum iCAM-1 levels, significantly higher serum VEGF levels were obtained in the low HDL-c patients' subpopulation, which might be a mechanism for promotion of endothelial repair under these circumstances.

Among the factors studies, some of them seem to have particular relevance, as the correlations analysis indicates. In fact, in the patients' population, but in particular in the subgroup with normal HDL-c levels, interesting correlations were found between three parameters (large HDL-c, Ox-LDL, and adiponectin) and several classical and nonclassical markers/risk factors. Large HDL-c contents showed a significant inverse correlation with Ox-LDL, TGs, non HDL-c and TNF-*α*, and direct with adiponectin. Ox-LDL values presented inverse significant correlation with large HDL-c and direct and significant with small HDL-c, TGs, TNF-*α*, and DBD. In addition, adiponectin concentrations presented statistically significant direct correlation with large HDL-c and inverse with waist circumference, hsCRP, uric acid, and TGs contents. These important associations were more evident for the normal HDL-c patients' subgroup and appear less associated in the subgroup with low HDL-c levels, which seem to indicate that when HDL-c levels are below the average values there is a deregulation of the factors (lipidic, oxidative, inflammatory, and angiogenic), with a putative important impact on the evolution of vascular disease.

Considering the cardiometabolic impact of low HDL-c levels on this type of patients with previous cardiovascular risk factors, even when LDL-c concentrations are adequately managed by antidyslipidemic therapy, therapeutic measures able to improve HDL-c levels and their quality/functionality, as well as inhibit LDL oxidation, might be of key importance to reduce the residual risk previously identified on this type of populations, namely, by reducing the oxidative, inflammatory, and angiogenic mechanisms underlying the evolution of disease. Since the current therapeutic arsenal is of limited impact on HDL-c levels, in particular the most popular medication, such as statins, nonpharmacological measures might deserve more attention, as well as new and more effective agents that might prove efficacy to improve HDL and their beneficial effects, including reduction of Ox-LDL as well as of deleterious inflammatory mediators. In fact, current data increasingly recommends aggressive measures to raise HDL-c levels and functionality as part of the prevention and treatment of CHD, while the novel HDL and Ox-LDL-directed pharmacotherapeutic strategies under discovering and evaluation will be able to help on this battle [22, 23, 62–64].

The study has some limitations that deserve further research in a near future: (a) the possibility of bias related with the inclusion of control subjects (defined as not having previous diagnosis for CV disease and not taking any medication for that) with risk factors, such as increased blood pressure and with overweight; (b) a more ample anthropometric and biochemical characterization would strength the results and findings; (c) the influence of other factors in this type of patients, such as menopausal status, lifestyle habits, and medication taken, will improve the data.

## 5. Conclusions

In a patient population with cardiovascular risk factors, low HDL-c levels are associated with a poor cardiometabolic profile, despite the average levels of LDL-c. This condition is better viewed by nontraditional lipid markers, including HDL subpopulations (in particular the large HDL-c one) and oxidized LDL, as well as markers of inflammation and angiogenesis, such as hsCRP, adiponectin, and VEGF. The existence of average HDL-c levels, with improvement of HDL quality/functionality, reduction of Ox-LDL and hsCRP, and increment of adiponectin, might prevent the evolution of cardiovascular disease in this type of individuals. In fact, despite called patients with residual cardiovascular risk, they often have non-fatal and fatal cardio and cerebrovascular events. Proper pharmacological and nonpharmacological therapeutic interventions directed to raise HDL-c levels and functionality and to inhibit Ox-LDL levels are advisory preventive measures in this type of CV risk populations.

## Figures and Tables

**Table 1:** Anthropometric data and general characterization of the study groups.

Parameters	Control group			Patients group		
	Normal HDL (*n* = 51)	Low HDL (*n* = 22)	*P*	Normal HDL (*n* = 119)	Low HDL (*n* = 50)	*P*
Age (years)	57.6 ± 8.3 [55.2–59.9]	57.9 ± 9.3 [53.8–62.0]	0.893	62.0 ± 9.9 (13.0)	60.0 ± 9.1 (14.0)	0.629
BMI (Kg/m^2^)	27.0 ± 3.6 [26.0–28.0]	28.8 ± 5.7 [26.2–31.3]	0.195	29.2 ± 4.8 [28.3–30.1]	29.9 ± 4.4 [28.6–31.1]	0.391
WC (cm)	96.5 ± 11.7 [93.1–99.8]	98.7 ± 12.9 [93.0–104.5]	0.468	103.5 ± 12.9 [101.0–105.9]	102.4 ± 13.5 [98.2–106.5]	0.644
SBP (mmHg)	140 ± 20 [135–146]	155 ± 21 [145–164]	0.007	140 ± 23 [136–144]	135 ± 22 [129–141]	0.164
DBP (mmHg)	84.9 ± 10.2 [82.0–87.9]	93.0 ± 10.3 [88.5–97.6]	0.003	78.0 ± 12.9 [75.7–80.4]	75.4 ± 13.6 [71.5–79.2]	0.234
Glucose (mmol/L)	5.42 ± 0.61 [5.24–5.59]	5.31 ± 0.45 [5.11–5.52]	0.494	6.38 ± 3.74 (4.86)	8.49 ± 4.12 (6.66)	0.228
HbA1c (%)	6.04 ± 0.49 [5.59–6.50]	6.30 ± 0.30 [5.55–7.05]	0.641	8.12 ± 1.92 [7.68–8.57]	9.38 ± 2.20 [8.58–10.17]	0.004

Results are presented as mean ± SD, lower and upper bound 95% confidence interval for mean, and *P* values obtained using independent samples *t*-test in the normal distribution samples and as median ± SD, interquartile range, and *P* values obtained using Mann-Whitney test in the non-normal distribution samples. BMI: body mass index; HbA1c: glycated hemoglobin; SBP: systolic blood pressure; DBP: diastolic blood pressure; WC: waist circumference.

**Table 2:** Lipid profile and markers of inflammation, angiogenesis, and endothelial lesion of the study groups.

Parameters	Control group			Patients group		
	Normal-HDL (*n* = 51)	Low-HDL (*n* = 22)	*P*	Normal-HDL (*n* = 119)	Low-HDL (*n* = 50)	*P*
Lipid profile						
Total-c (mmol/L)	5.67 ± 0.92 [5.41–5.93]	5.17 ± 0.83 [4.80–5.53]	0.030	4.88 ± 1.07 [4.68–5.07]	5.13 ± 1.14 [4.81–5.46]	0.175
TGs (mmol/L)	1.07 ± 0.37 [0.96–1.17]	1.47 ± 0.50 [1.25–1.69]	0.000	1.24 ± 0.84 (0.90)	2.32 ± 1.31 (2.24)	0.000
Total HDL-c (mmol/L)	1.58 ± 0.30 [1.49–1.66]	1.10 ± 0.16 [10.3–1.17]	0.000	1.48 ± 0.30 [1.42–1.53]	1.01 ± 0.17 [0.97–1.06]	0.000
Large HDL-c (%)	34.7 ± 14.4 (13.1)	31.5 ± 11.3 (11.4)	0.176	35.1 ± 11.8 [32.9–37.2]	27.5 ± 11.1 [24.3–30.6]	0.000
Interm HDL-c (%)	46.5 ± 7.6 (6.9)	48.3 ± 5.6 (7.1)	0.133	45.4 ± 6.3 (8.1)	48.7 ± 7.1 (6.4)	0.000
Small HDL-c (%)	18.2 ± 9.00 [15.7–20.7]	18.5 ± 7.9 [15.0–22.0]	0.886	19.8 ± 8.2 [18.3–21.3]	24.5 ± 11.9 [21.2–27.9]	0.001
LDL-c (mmol/L)	3.60 ± 0.90 [3.35–3.85]	3.40 ± 0.80 [3.04–3.75]	0.359	2.74 ± 0.95 [2.56–2.91]	3.02 ± 1.00 [2.73–3.31]	0.096
Ox-LDL (U/L)	45.7 ± 18.7 [40.4–51.1]	39.2 ± 11.5 [34.1–44.3]	0.202	35.7 ± 12.7 [33.2–38.2]	40.1 ± 14.2 [35.6–44.5]	0.043
Ox-LDL/LDL-c	12.6 ± 3.9 [11.5–13.8]	11.7 ± 3.0 [10.4–13.1]	0.344	13.1 ± 3.5 [12.4–13.8]	14.00 ± 4.1 [12.7–15.3]	0.300
Non-HDL-c (mmol/L)	4.10 ± 0.94 [3.83–4.36]	4.07 ± 0.83 [3.70–4.44]	0.915	3.40 ± 1.08 [3.21–3.60]	4.12 ± 1.11 [3.80–4.44]	0.000
Total-c/HDL-c	3.72 ± 0.91 [3.47–3.98]	4.81 ± 1.07 [4.33–5.28]	0.000	3.42 ± 0.96 [3.25–3.60]	5.18 ± 1.34 [4.80–5.56]	0.000
LDL-c/HDL-c	2.39 ± 0.80 [2.16–2.61]	3.17 ± 0.96 [2.74–3.59]	0.001	1.94 ± 0.80 [1.79–2.08]	3.04 ± 1.16 [2.70–3.38]	0.000
PON1 activity	505 ± 131 [469–542]	443 ± 109 [394–491]	0.042	494 ± 173 [462–525]	510 ± 236 [443–578]	0.774

Markers of inflammation, angiogenesis, and endothelial lesion						
hsCRP (*µ*g/mL)	0.25 ± 0.36 [0.13–0.37]	0.37 ± 0.44 [0.11–0.64]	0.103	0.22 ± 0.54 (0.41)	0.50 ± 0.56 (0.71)	0.034
TNF-*α* (pg/mL)	3.56 ± 3.23 [2.65–4.48]	3.29 ± 3.35 [1.80–4.78]	0.880	3.12 ± 2.69 [2.59–3.66]	3.28 ± 2.53 [2.49–4.07]	0.560
Adiponectin (*µ*g/mL)	10.8 ± 6.9 [8.9–12.8]	8.0 ± 5.5 [5.6–10.5]	0.069	8.9 ± 6.6 [7.6–10.2]	7.1 ± 5.6 [5.3–8.9]	0.041
Uric acid (mmol/L)	0.32 ± 0.09 [0.30–0.35]	0.29 ± 0.10 [0.24–0.35]	0.283	0.36 ± 0.38 (0.44)	0.40 ± 0.39 (0.59)	0.539
VEGF (pg/mL)	385 ± 388 [274–497]	408 ± 329 [262–554]	0.531	405 ± 295 [346–464]	520 ± 321 [420–620]	0.019
iCAM-1 (ng/mL)	413 ± 318 (160)	486 ± 586 (176)	0.122	572 ± 216 [512–631]	471 ± 137 [411–532]	0.070

Results are presented as mean ± SD, lower and upper bound 95% confidence interval for mean, and *P* values obtained using independent samples *t*-test in the normal distribution samples and as median ± SD, interquartile range, and *P* values obtained using Mann-Whitney test in the non-normal distribution samples. CRP: C-reactive protein; HDL-c: high-density lipoprotein cholesterol; iCAM-1: intercellular adhesion molecule 1; LDL-c: low-density lipoprotein cholesterol; Ox-LDL: oxidized low-density lipoprotein; TGs: triglycerides; TNF-*α*: tumour necrosis factor alpha; Total-c: total cholesterol; VEGF: vascular endothelial growth factor.
